# Early monitoring‐to‐warning Internet of Things system for emerging infectious diseases via networking of light‐triggered point‐of‐care testing devices

**DOI:** 10.1002/EXP.20230028

**Published:** 2023-10-05

**Authors:** Yu Fu, Yan Liu, Wenlu Song, Delong Yang, Wenjie Wu, Jingyan Lin, Xiongtiao Yang, Jian Zeng, Lingzhi Rong, Jiaojiao Xia, Hongyi Lei, Ronghua Yang, Mingxia Zhang, Yuhui Liao

**Affiliations:** ^1^ Molecular Diagnosis and Treatment Center for Infectious Diseases Dermatology Hospital Southern Medical University Guangzhou China; ^2^ Longgang District Central Hospital of Shenzhen Shenzhen China; ^3^ National Clinical Research Center for Infectious Disease the Second Affiliated Hospital of Southern University of Science and Technology Shenzhen Third People's Hospital Shenzhen China; ^4^ Institute for Health Innovation and Technology National University of Singapore Singapore Singapore; ^5^ Department of Burn Surgery the First People's Hospital of Foshan Foshan China; ^6^ Department of Burn and Plastic Surgery Guangzhou First People's Hospital South China University of Technology Guangzhou China

**Keywords:** molecular diagnostics, monitoring and warning system, pathogen identification, point‐of‐care testing, sample to answer

## Abstract

Early monitoring and warning arrangements are effective ways to distinguish infectious agents and control the spread of epidemic diseases. Current testing technologies, which cannot achieve rapid detection in the field, have a risk of slowing down the response time to the disease. In addition, there is still no epidemic surveillance system, implementing prevention and control measures is slow and inefficient. Motivated by these clinical needs, a sample‐to‐answer genetic diagnosis platform based on light‐controlled capillary modified with a photocleavable linker is first developed, which could perform nucleic acid separation and release by light irradiation in less than 30 seconds. Then, on site polymerase chain reaction was performed in a handheld closed‐loop convective system. Test reports are available within 20 min. Because this method is portable, rapid, and easy to operate, it has great potential for point‐of‐care testing. Additionally, through multiple device networking, a real‐time artificial intelligence monitoring system for pathogens was developed on a cloud server. Through data reception, analysis, and visualization, the system can send early warning signals for disease control and prevention. Thus, anti‐epidemic measures can be implemented effectively, and deploying and running this system can improve the capabilities for the prevention and control of infectious diseases.

## Graphical abstract

There is still no epidemic surveillance system, implementing prevention and control measures is slow and inefficient. This study developed a rapid point‐of‐care (POC) testing device for detecting pathogens. Through multi‐device networking, an early monitoring and warning system for major infectious diseases was developed. This study makes a contribution to the urgent need for a system to prevent and control the spread of epidemic diseases.

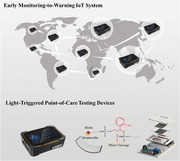

## 1 INTRODUCTION

In recent years, new infectious threats have emerged almost yearly,^[^ ^1^ ^]^ such as dengue fever disease in 1998,^[^ ^2^ ^]^ Ebola virus disease in 2002,^[^ ^3^ ^]^ and SARS‐CoV in 2003.^[^ ^4^ ^]^ In 2019, SARS‐CoV‐2 emerged and spread globally, infecting more than 500 million people and causing over 6 million deaths.^[^ ^5^ ^]^ These diseases present the characteristics of multiple sources, rapid transmission, and widespread infection, which has brought great challenges to preventing and controlling infectious diseases and social stability.^[^ ^6^ ^]^ Therefore, early monitoring and warning platforms for major infectious diseases are becoming increasingly important. Currently, data collected for disease prevention systems only come from the diagnosis results of medical institutions and the reports of disease control departments. The limitations of the existing system include the low number of data acquisition sources, long response time, and lack of early warning ability.^[^ ^7^ ^]^ This study focused on improving detection and warning capabilities in the early stages of disease transmission.

To the best of our knowledge, the key to solving these problems is to construct an accurate and timely point‐of‐care (POC) testing platform so that on‐site data can be collected promptly.^[^ ^8^ ^]^ A vast array of portable devices can make up the Internet of Things (IoT) for medical testing to increase the sources of disease information and improve the average response time.^[^ ^9^ ^]^ Thus, the early monitoring and warning of infectious threats can be achieved. Currently, polymerase chain reaction (PCR) and derivative technologies,^[^ ^10^ ^]^ with unparalleled sensitivity and specificity, are considered as the gold standard for the early diagnosis of infectious pathogenic microorganisms. However, the entire procedure, including sample pretreatment, nucleic acid (NA) extraction, amplification, and detection, relies on high‐end equipment and skilled personnel.^[^ ^11^ ^]^ These factors limit the widespread application of nucleic acid amplification techniques (NAATs) in resource‐limited environments. There has been intensive research on the design of an automated detection platform with ‘Sample‐to‐Results’ integration. For instance, Biofire FilmArray performed multiplex PCR in 60 min.^[^ ^12^ ^]^ AlereI could detect influenza A&B viruses in 15 min with 25−97.4% sensitivity.^[^ ^13^ ^]^ Although significant advances have been made, there is still a lack of an ideal tool for POC testing, which should be sensitive, rapid, low‐cost, and easy to use by minimally trained personnel.^[^ ^14^ ^]^

In this work, a sample‐to‐answer nucleic acid detection system was developed for POC testing of infectious diseases. In this system, a novel light‐triggered capillary is used for rapid NA isolation and purification, and a handheld convective PCR device is integrated to perform downstream amplification and detection.^[^ ^15^ ^]^ To achieve this, a photocleavable (PC) linker was used to connect the inner surface of the silica capillary to the polymer layer with a positive charge. Thus, the negatively charged NA can be absorbed and separated through electrostatic adsorption. After inhaling the PCR reagent, the capillary was irradiated with 365 nm UV light. The PC linker can be cut off based on the photochemistry of *o*‐nitrobenzyl ethers to allow the genomic DNA to be released into the liquid.^[^ ^16^ ^]^ By assembling the capillary in a thermal gradient system, on‐site convective PCR amplification can be achieved without active cooling or heating. This design significantly simplifies the structure of the device. Moreover, it can be used for POC testing as an easy‐to‐operate method. Practical samples from COVID‐19 patients were tested to evaluate the effectiveness of this system. This system, integrated with the functions of novel solid‐phase NA extraction and rapid PCR amplification, allows for fast, user friendly and robust infectious diseases diagnosis.

Moreover, artificial intelligence analysis for real‐time surveillance data of pathogens combining different input factors can provide managers and non‐experts with decision‐making and activity guidance.^[^ ^17^ ^]^ It is functional in guiding public health policymaking. For example, Feng et al. used the Susceptible‐Exposed‐Infectious‐Removed (SEIR) model to predict COVID‐19 epidemic peaks and sizes.^[^ ^18^ ^]^ Huang's group developed a Global Prediction System for the COVID‐19 Pandemic that is qualified for the forecasting and early warning of disease transmission.^[^ ^19^ ^]^ However, some issues still need to be addressed, such as the limited data acquisition sources and long response times.

**FIGURE 1:**
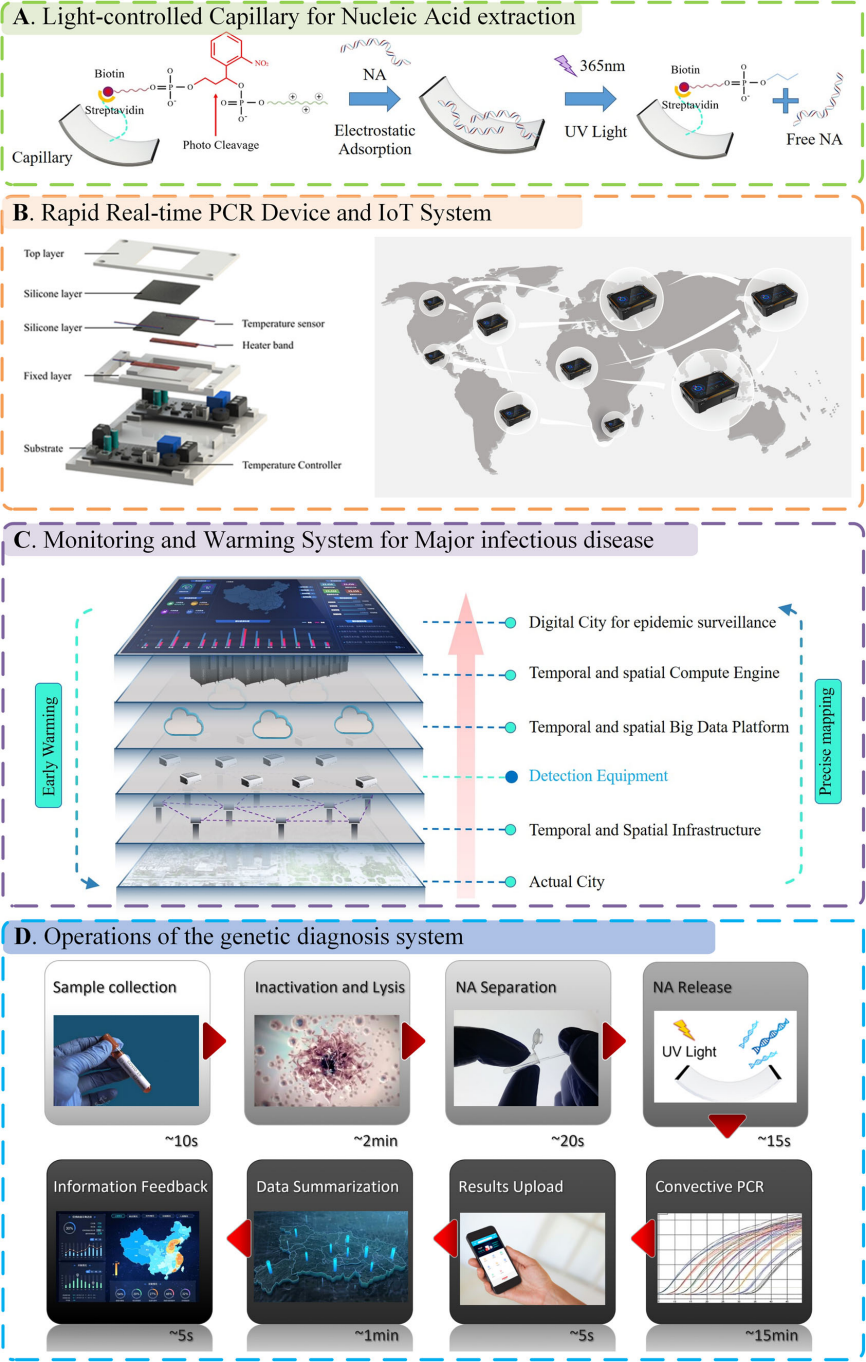
Schematic of the genetic diagnosis method and the monitoring and warning system. A the principle of the light‐controlled capillary for nucleic acid (NA) extraction; B the exploded view shows the architecture and components of the convection polymerase chain reaction (PCR) device and the construction of Internet‐of‐Things (IoT) system; C construction of the monitoring and warming system. D operations of the genetic diagnosis system and the time required for each step.

Here, we developed an early monitoring and warning platform for the detection of major infectious diseases. This system was realized using a combination of software and hardware. The hardware components included a portable and rapid NA diagnosis platform, personnel information identification module, and fifth‐generation (5G) communication module. These pieces of equipment located in different areas form the Internet of Things. The detection results, bound to the personnel information, can be uploaded to the monitoring and warning platform. The software was developed on a cloud server and was responsible for data reception, analysis, and visualization. According to the regional pathogen detection data, we can divide areas with different levels of transmission risk, and the early warning signal will be fed back to the individuals and centres for disease control and prevention. Thus, these measures can be performed effectively and promptly. In order to predict the restricted area, we design an artificial intelligence model for prediction of the emerging or new infectious diseases. The positive patient activity trajectories and the corresponding restricted areas which are collected by government are used for model computation. Core concept of the model is used the earlier, already happened samples to training the model, then use the trained model for prediction. In the application process, the positive patient activity trajectories are fed into the model and then the advised restricted area are provided by the model automatically. The system ran stably and reliably, and the research results provided a reference for preventing and controlling infectious diseases. A schematic of the genetic diagnosis method and monitoring and warning system is shown in Figure 1A–C.

## 2 RESULTS AND DISCUSSION

### 2.1 Operation of the system

In this study, the complete system contained a rapid detection unit and a software platform for epidemic monitoring and warning. The hardware unit had three fundamental functions: (1) NA separation and purification, (2) light‐triggered NA release, and (3) convection PCR amplification. As shown in Figure 1D, the cultured bacteria, diluted in deionized water, was heated to 95°C for 5 min to release the NA. The lysate was then introduced into a light‐controlled capillary by the capillary and siphon force. The PC linker was used to connect the inner surface of the silica capillary to the polymer layer with a positive charge. Thus, with electrostatic forces, the negatively charged NA was absorbed on the inner surface of the capillary with positively charged. After 20 s, the lysate flowed out under the action of gravity. Next, the regent used for PCR amplification was introduced into capillaries. All were sealed and assembled in the thermal‐gradient scaffold for succeeding convection PCR amplification and detection.

Before NA amplification, the light‐controlled capillaries were exposed to UV light at 365 nm for 15 s to release the NA by light irradiation irreversibly. The photocleavability principle was based on the photochemistry of *o*‐nitrobenzyl ethers, therefore, the NA can be released back into the liquid. Then, one side of the scaffold was heated to 95°C, and the thermal conduction of the substrate passively heated the other side. This could establish a desirable thermal gradient along the silicone surface, which was well applied to perform real‐time amplification. Fluorescence images were obtained in the fluorescence detection zone, where a good linear thermal gradient was formed. Then the software measured and recorded the fluorescence intensity in the capillaries, so that the qPCR curves were generated as the final test results.

Information about the person being tested, including the detection result, name, age, gender, identity card (ID) number, home address, and places visited up to seven days before, was uploaded to the early monitoring and warning platform by the built‐in 5G communication module. Based on this information, the system manager can mark areas with different risk levels. The reports of epidemic diseases and warning signals as evidence for the development of prevention and control measures were fed back to the local medical facilities in a timely manner. Simultaneously, a warning signal was issued in the WeChat small program to warn users to stay away from risk areas.

### 2.2 Synthesis of the photocleavable linker and evaluation of photolysis

**FIGURE 2:**
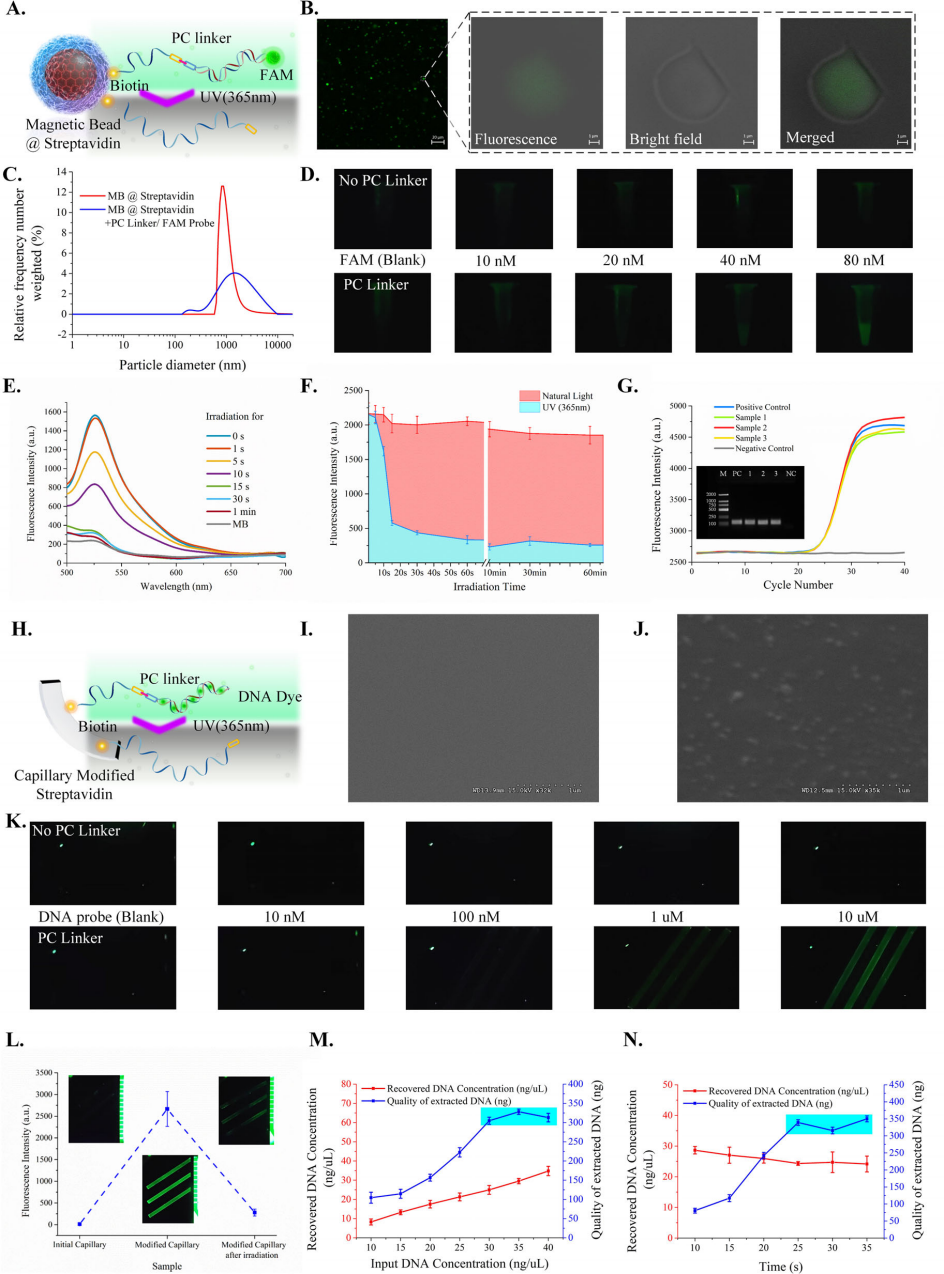
Synthesis of the photocleavable linker and evaluation of the photolysis. A synthesis of photocleavable (PC) linkers on the surface of magnetic beads; B confocal microscope image of the magnetic beads with PC linkers and FAM‐ssDNA probe; C the particle size of the magnetic beads before and after modification; D the fluorescence image of magnetic beads (with/without PC linkers) modified FAM‐ssDNA probe with different concentration; E cleavage efficiency of the PC linker; F contrast of cleavage results irradiated by natural light and ultraviolet (UV) light; G amplification results of the NAs irradiated by UV light; H synthesis of PC linkers on the surface of the capillaries; I Scanning electron microscope (SEM) image of bare silica wafer; J SEM image of streptavidin and PC linker coated silica wafer; K the fluorescence image of capillaries (with/without PC linkers) modified DNA Dye with different concentration; L validation of the synthesis and working process; M capacity for NA separation at different times; (N) quality of extracted NAs from the input sample at different concentrations.

To quantitatively analyze the photolysis efficiency, we first modified the PC linker on the surface of the magnetic beads. A schematic of the synthesis and photolysis process is shown in Figure 2A. The PC ssDNA sequence modified with biotin at the 3′ end was connected to magnetic beads modified with streptavidin. Then, the complementary sequence modified with carboxyfluorescein (FAM) at the end of 5′ was incubated for 1 min. After washing out the superfluous ssDNA with FAM, the fluorescence signal could be read only on the surface of the magnetic beads.

As shown in Figure 2B, the obvious fluorescence signals on the surface of the magnetic beads could be read from the confocal microscope images with super‐resolution. The changes in particle diameter after modification are also shown in Figure 2C. As shown in Figure 2D, the fluorescence signals of the magnetic beads modified with the PC linker were enhanced as the concentration of ssDNA with FAM increased. These results proved that the PC linker and ssDNA probe with FAM were successfully modified on the surface of the magnetic beads. We irradiated the magnetic beads with UV at 365 nm for different times so that a part of the dsDNA modified with FAM was cut off. After washing with ddH_2_O, the fluorescence intensity was detected, as shown in Figure 2E. The results showed that the fluorescence intensity decreased significantly with increasing irradiation time. The cleavage of the PC linker was completed in 15 s. Next, we tested the efficiency of photolysis under natural light. As shown in Figure 2F, there was no significant weakening of fluorescence intensity after irradiation with natural light for 60 min. These results demonstrated that this method could be used for POC testing in the field. In addition, we irradiated genomic DNA extracted from *Staphylococcus aureus* (*Sta*) and used it as a target for PCR amplification. The results are shown in Figure 2G, which demonstrates that UV light at 365 nm cannot cause damage to NA, and this method can be integrated with downstream amplification and detection processes.

We then determined the functionalization process of the light‐controlled capillaries. The modification of streptavidin on the surface of the capillary was described above. As shown in Figure 2H, the PC ssDNA sequence modified with biotin at the 3′ end was first connected to the capillary modified with streptavidin. The other complementary sequences were incubated for 1 min. SYBR Green I, a fluorescent dye, was bound to the dsDNA.

Scanning electron microcopy (SEM) images are shown in Figure 2I, and the surface of the bare silica wafer was smooth. Its surface became uneven when the wafer was modified with streptavidin and a PC linker (Figure 2J). These results demonstrate the feasibility of this synthesis scheme. The inner surface of the capillaries was then modified using the same method. The results are shown in Figure 2K; as the concentration of the complementary sequences and DNA dye increased, the fluorescence signals of the capillaries modified with streptavidin and the PC linker were enhanced. Additionally, as shown in Figure 2L, the initial capillaries exhibited no fluorescence signals. After modification, the capillaries exhibited clear and strong fluorescent signals. Then, the capillaries were irradiated with UV at 365 nm for 15 s and washed with ddH_2_O, and the intensity of the fluorescence signals decreased simultaneously. These results verified the successful modification of the PC linkers. The poly diallyldimethylammonium chloride (PDDA) modification has been described in our previous work. This modification changed the potential of the capillaries, so that we could use the capillaries to absorb NA by electrostatic forces.

We then evaluated the capillaries for DNA extraction. A total of 60 μL of genomic DNA, with initial concentrations ranging from 10 to 40 ng μL^−1^, was introduced into the capillaries. After incubation for the 60 s and irradiation with UV light, the sample was recovered, and the concentration of residual genomic DNA was determined. As shown in Figure 2M, the quality of the extracted DNA increased until the concentration of the input DNA was higher than 30 ng μL^−1^. This result proved that the maximum capacity for DNA extraction in light‐controlled capillaries was approximately 320 ng. Next, 60 μL genomic DNA at an initial concentration of 30 ng μL^−1^ was introduced into the capillaries. After incubation for a certain time (from 10 to 35 s), the genomic DNA was released, and the concentration of the recovered sample was tested. As shown in Figure 2N, the genomic DNA absorbed on the inner surface of the capillary was saturated when the detention time was longer than 25 s. Thus, the light‐controlled capillaries could perform NA extraction in less than 25 s, and the NA extraction time was less than 20 s. In this study, we developed a simple and rapid method for extracting, separating, and releasing NA.

### 2.3 Evaluation of thermal management and quantitative detection on the system

**FIGURE 3:**
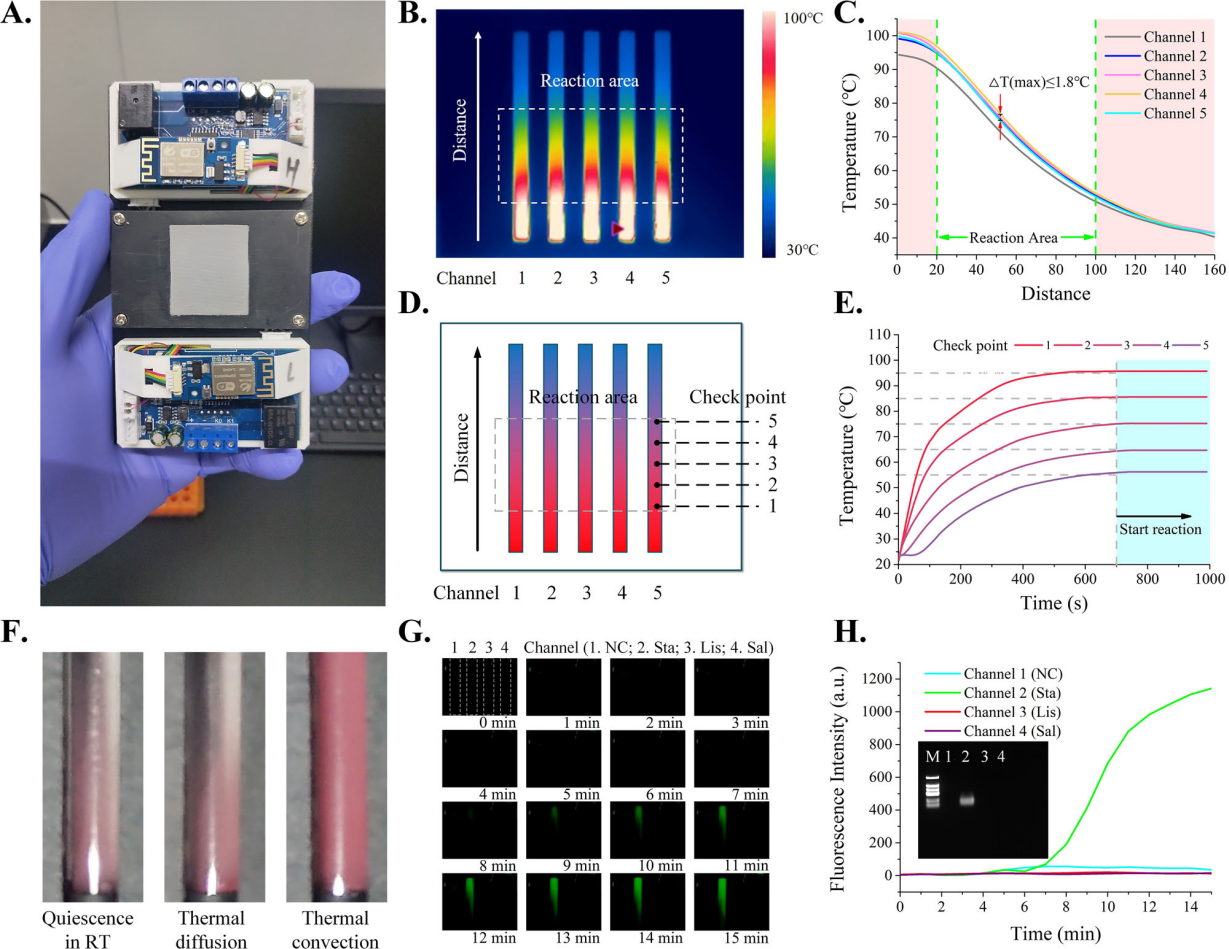
Evaluation of temperature control and real‐time PCR detection on the system. A photograph of the rapid real‐time PCR device; B infrared thermal imaging of the thermalization chamber for PCR amplification; C temperature of each channel; D the distribution of five temperature measuring points in one channel; E thermal time‐responses of five representative points; F the movement of polystyrene beads in the capillaries under different conditions; G fluorescence images of the capillaries during the amplification process; H amplification curves from this system.

Because temperature is one of the key factors to ensure the PCR specificity and sensitivity, we first evaluated the stability of thermal convection in the reaction chambers. A photograph of the convection PCR device is presented in Figure 3A. Infrared images of the thermal gradient scaffold are shown in Figure 3B. When one side of the scaffold was heated to 95°C, the other side was passively heated by the thermal conduction of the substrate, and a uniformly distributed temperature gradient was formed. To verify the temperature distribution, we measured the temperature at different locations; the results are shown in Figure 3C. In the reaction area, a desirable thermal gradient ranging from 60°C to 95°C was formed along the lengthwise direction of the substrate. The maximum temperature difference in the reaction channels was less than 1.8°C. The temperature of thermal reflection area in the device was stable and uniform, so that the PCR amplification can be performed effectively. In addition, we measured the temperature at five points evenly distributed in one channel. As shown in Figure 3D,E, a temperature with high stability at all checkpoints was obtained after the heating process, which lasted approximately 10 min. Polystyrene beads sealed in the capillaries were used to observe the thermal convection phenomenon. As shown in Figure 3F, polystyrene beads settled at the bottom of the capillary at room temperature. The capillaries were heated to the specified temperature and placed horizontally, and the state of the polystyrene beads did not change under the action of thermal diffusion. Then, we installed the capillary on the heating module with a temperature gradient and placed it vertically. With thermal convection, the polystyrene beads moved and were distributed uniformly in the water phase.

To characterize the ability of this system for rapid and simultaneous DNA amplification and detection, convective PCR assays were carried out to detect *Sta*. *Listeria monocytogenes* (*Lis*) and *Salmonella enteric* (*Sal*) were used as the control group to verify the test specificity. Figure 3G shows the fluorescence signals of the amplicons over 15 min. The fluorescent images were taken in one‐minute segments, and we can see that the signals intensity began to increase from the eighth minute. The fluorescence signal intensity was measured and the amplification curves were drew as shown in Figure 3H. Agarose gel electrophoresis was performed as auxiliary evidence of detection accuracy. These results suggest that the present system can be used for the simultaneous identification of multiple samples, and it has the potential for simple, rapid, and quantitative DNA analysis.

### 2.4 Demonstration of NA detection with practical samples from COVID‐19 patients

**FIGURE 4:**
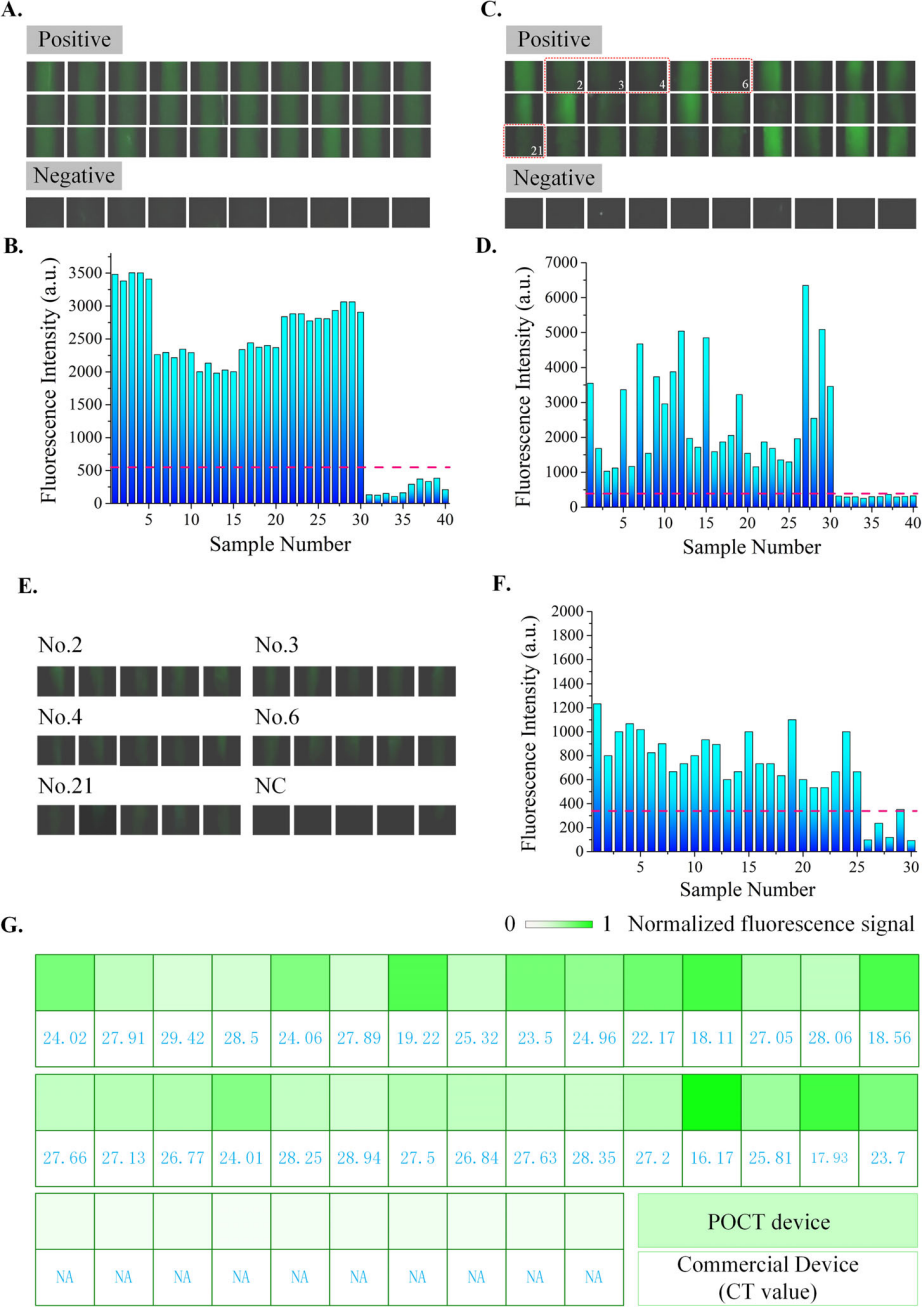
Demonstration of DNA extraction and detection with practical samples. A Fluorescence images of the synthetic N‐gene fragment as a standard sample; B the fluorescence intensity of each sample; C test results of 50 clinical nasopharyngeal swab RNA extracts; D fluorescence intensity of each sample; E repeated experiment results of five practical samples with low target concentration; F fluorescence intensity of each sample; G Comparison of detection results from our device and commercial device.

First, we synthesized the N‐gene fragment of SARS‐CoV‐2 as a standard positive control to verify the feasibility of the prototype device. Driven by capillary and siphon force, the sample was introduced into a light‐controlled capillary, and after 20 s, the sample flowed out under the action of gravity. The capillaries filled with PCR mix were then sealed. These were assembled in a convective system. After exposure to UV light at 365 nm for 15 s, the convective system was heated to the specified temperature for PCR amplification. A charge coupled device (CCD) camera captured the fluorescence images of 40 positive samples and 10 negative samples in the 20th min. Fluorescence images are shown in Figure 4A. The fluorescence intensity of each sample was accurately measured, and quantitative information is shown in Figure 4B. These test results indicate that this method is feasible for detecting a standard‐positive SARS‐CoV‐2 sample.

We then used the detection platform to analyze RNA extracts from 50 clinical nasopharyngeal swabs. Swab specimens were collected from 40 confirmed cases and 10 healthy individuals. The detection process is described above, and the fluorescence images are shown in Figure 4C. The fluorescence intensity of each sample was measured, as shown in Figure 4D. The fluorescence signals of 40 COVID‐19‐positive samples were clearly distinguished from those of the negative samples. To further verify our method's stability, five clinical samples with low target concentrations were selected to repeat the experiment. As shown in Figure 4E,F, all positive samples were successfully detected. A comparison of the detection results from our device and a commercial device is shown in Figure 4G. Thus, we anticipate using this method for rapid POC testing in clinical settings.

### 2.5 Design and implementation of the early monitoring and warning platform for major infectious diseases

**FIGURE 5:**
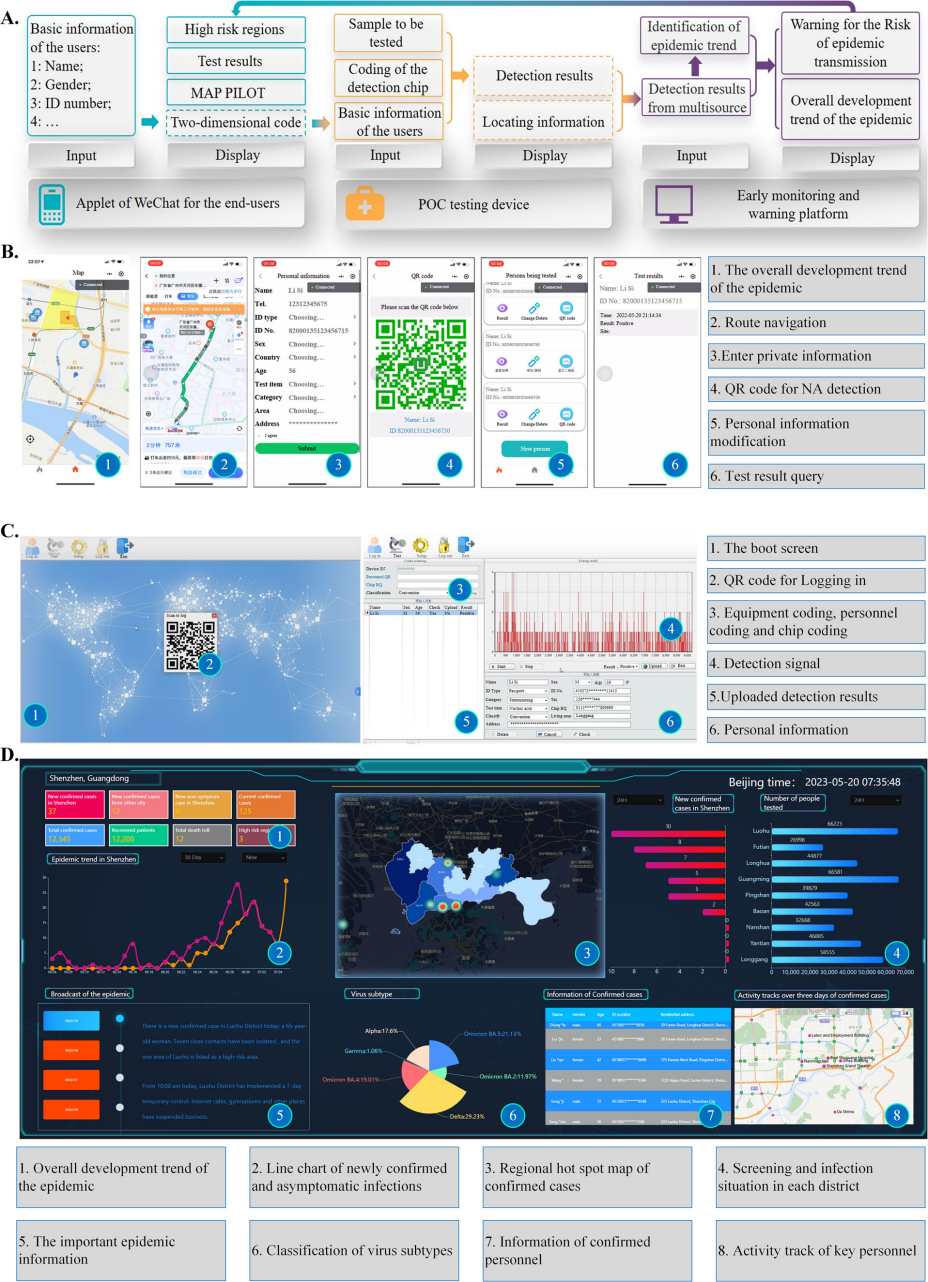
Main functions of the monitoring and warning system. A The overall system structure of the monitoring and warning platform; B operation interface of the applet in WeChat; C User interface (UI) of the point‐of‐care (POC) testing device; D overall content of the early monitoring and warning platform.

The overall system structure of the monitoring and warning platform is shown in Figure 5A, which comprises three main subsystems: an applet of WeChat for the end‐users, detection software on the POC testing device, and the monitoring and warning program on the cloud server.

By searching and opening the applet in WeChat, we could easily find the nearby detection stations on the built‐in map, and the areas with different levels of transmission risk were also marked on the maps, as shown in Figure 5B. Route navigation led us to detection stations. Before NA testing, we needed to register basic information on the applet, such as name, gender, ID number, age, and home address. An exclusive two‐dimensional code was generated for the subsequent detection. We can also view the previous detection results for this applet.

When the POC testing device was started, the login user interface (UI) was presented, as shown in Figure 5C. Only medical staff can log in to their accounts using passwords. Before the test, people waiting for NA testing need to show their two‐dimensional code. The personal details can be read out by the code scanner integrated into the device and displayed at the interface of the detection software. In addition, the capillary used for detection also had a unique ID that was bound to personal details. After confirmation, the medical staff performed NA detection. The results were produced in less than 20 min and uploaded to the monitoring and warning program on the cloud server by the 5G communications module.

The front‐end and back‐end systems of the early monitoring and warning platforms were developed based on Spring Boot and Vue architectures, respectively. The operating environment is a cloud server with a dual‐core 2 GHz CPU, 16 GB memory, 500 GB hard disk, and Ubuntu 18.10 and above operating system. JDK 1.8, Mysql 5.7.0, Redis 3.0, Maven 3.6, and other software packages were preinstalled on the server. The collected detection data from multiple sources were uploaded to the server and visualized, allowing the administrator to observe the development of the epidemic. The overall data visualization is shown in Figure 5D. Based on the administrative distinction, this platform showed reports of epidemic diseases on different spatial scales. Here, we consider Shenzhen as an example to describe the platform's main functions. This platform was mainly composed of the development trend of the epidemic, screening, and infection situation in each district, broadcasting of important epidemic information, classification of virus subtypes, information of confirmed personnel, and the activity tracking of key personnel. The overall development trend of the epidemic displayed the data of new confirmed cases, imported cases, asymptomatic local infections, existing confirmed cases, cumulative confirmed cases, cumulative cured cases, cumulative deaths, and risk areas on the same day. At the same time, a line chart and regional hot spot map of newly confirmed and asymptomatic local infections at 30 days were generated, and the epidemic information in Shenzhen was fed back in real‐time. The screening and infection situation showed the number of people tested and new confirmed cases in each district in 24 h to determine the key areas of concern. The important epidemic information broadcasting module broadcasts key epidemic information in Shenzhen in real‐time. The classification of virus subtypes showed the types and proportions of existing virus subtypes, which were used to evaluate the transmissibility of the virus and to formulate corresponding prevention and control strategies. The information on confirmed personnel and the activity tracking of key personnel shall be published in time to facilitate the rapid investigation of the possibility of close contact by the masses and to reduce the pressure of epidemic prevention and control. In summary, according to the requirements of reliability, real‐time performance, and comprehensiveness of the epidemic prevention and control system, an intelligent data visualization platform was developed to show information about COVID‐19 in a useful way for decision‐makers. Thus, the early warning signal is fed back to the individual and the disease control and prevention centres. Therefore, these measures can be performed effectively and in a timely manner.

### 2.6 Artificial intelligence model for prediction of the emerging or new infectious diseases

As conventional approaches require complicated model designs with parameter settings, deep learning offers a more efficient solution for viral forecasting, which is affected by many factors, including weather, public transit systems, population size, and some causal reasons. Here, we preliminarily presented a predictive model based on artificial intelligence for forecasting epidemic trends of emerging or new infectious diseases, such as COVID‐19. Here, we only considered Shenzhen's public transit system and population size for the model design.

The proposed predictive model was a supervised algorithm. The sample data comprised the positive patients’ epidemiological survey data obtained from Shenzhen Third People's Hospital. We supposed that public transportation is well‐established, human traffic is normal, and other factors such as weather and time are not considered. Based on the epidemiological survey data of positive patients, the activity trajectories of these individuals were expressed by their dates and addresses. For example, denoting A as a positive patient, data from the last seven days were recorded. The data are expressed as *d*_*i* (*i* = 1, 2, …,7), the pass‐through subway stations or bus stations are expressed as *S*_*n* (*n* = 1, 2, …), and the staying staff gathering places are expressed as *P*_*m* (*m* = 1, 2, …). Moreover, all the recorded locations were expressed as position coordinates. As a result, the sample data can be expressed as (*d, p*), where d denotes the date *d_i*, and *p* denotes the position coordinates (*x, y*), which are the coordinates of *S_n* and *P_m*. The restricted areas expressed by (*x, y, r*) that were caused by the positive patients were also obtained as labels, where *x* and *y* denote the restricted area, and *r* denotes the zone radius. As of now, we have completed the construction of sample data and labels.

**FIGURE 6:**
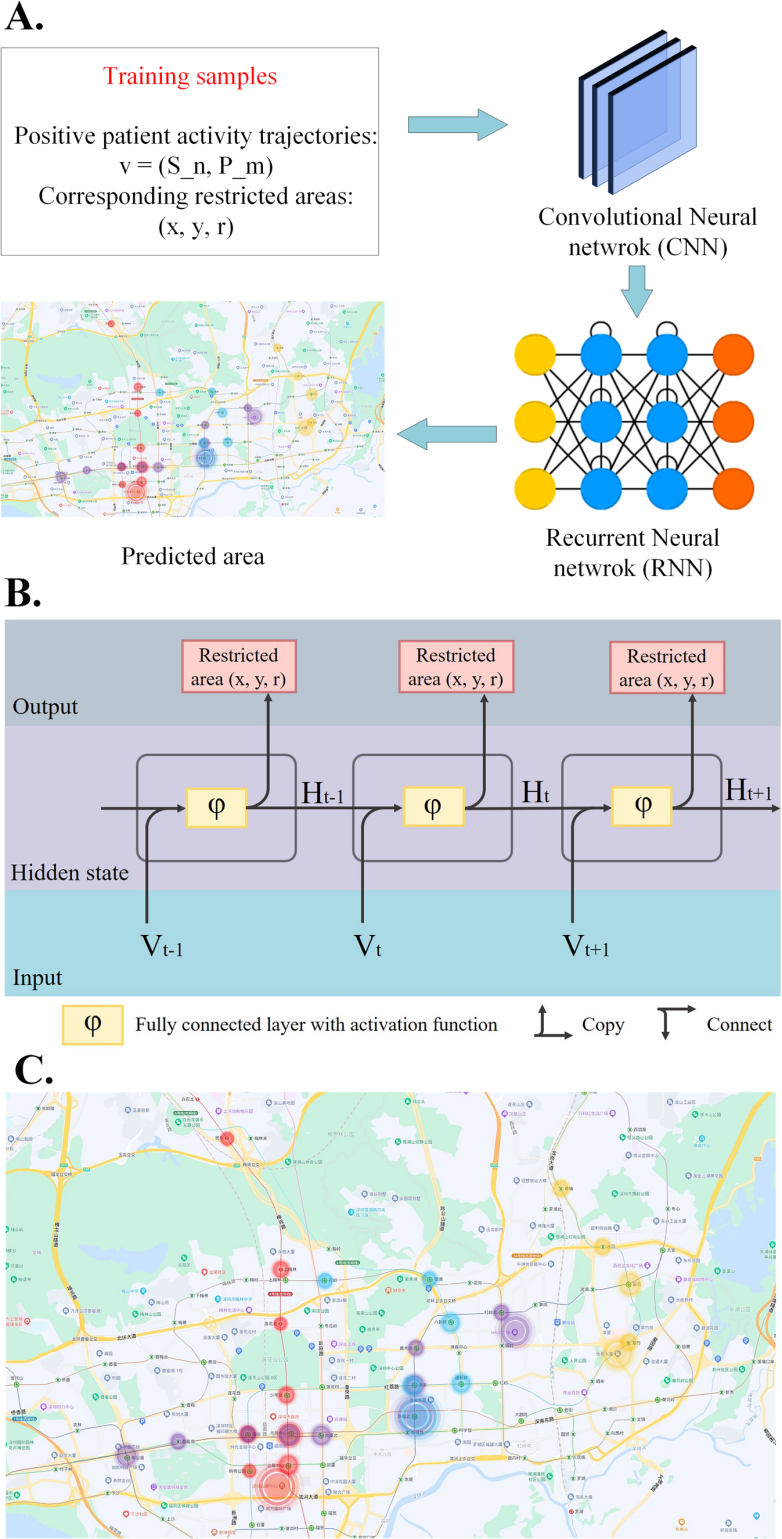
Artificial Intelligence model for prediction of the emerging or new infectious diseases. A The overview of the designed artificial intelligence (AI) model; B The fundamental structure of the recurrent neural networks (RNN) model; C the output plot of the model.

We design a deep learning model based on recurrent neural networks (RNN) for the prediction of restricted areas because the positive patients’ activity trajectories are time‐sequence data. The model is composed of training and testing processes, and the block diagram representation of the model is shown in Figure 6A,B, in which *x* and *y* represent the longitude and latitude of the metro station or the presence and movements of the patients, respectively, and *r* is the radius of influence. During the training process, positive patient activity trajectories *v* = (*S_n, P_m*) and the corresponding restricted areas (*x, y, r*) are input into the designed RNN for iterative computations.

The proposed network is composed of convolutional layers and recurrent layers, the first three convolutional neural networks which followed Rectified Linear Unit (ReLU) activation function and average pooling layers are used for feature extraction from the input data, then recurrent neural networks with five hidden layers are used for prediction. In the RNN, extracted data are input into the hidden layers, the input data of the next layer is composed of the raw extracted features and the prior layer's output. The learning rate of the network are set as 0.1, and the loss function is designed based on Euclidean distance.

This process ended when the number of iterations met this requirement. During the test, a positive patient activity trajectory was input into the trained model to predict the corresponding restricted area. As shown in Figure 6C, all predicted restricted areas were collected as Supplementary Information for government decisions. The computation time of a single dataset was less than 3 s.

The proposed model has the advantage of passing through complicated parameter settings, resulting in a dynamic correction. Intuitively, the trace of a random person and the corresponding affluence is a time‐related process, the RNN uses consecutive hidden layers to compute the sequential relation through the time‐axis. During training, high‐level features are output from convolutional neural network and the generated data is input into the recurrent neural network, the invariant feature of the raw data is retained automatically through the network. However, it is a simple predictive algorithm with limited forecast accuracy. In the future, we will improve the model by considering as many influencing factors as possible to design a multi‐input multi‐output model to predict emerging or new infectious diseases.

## 3 CONCLUSION

In summary, the simple, rapid, and quantitative POC testing platform of NA enables infectious disease diagnosis in resource‐limited settings. Connected to a PC linkage molecule, the positively charged polymer layer on the capillaries was used for NA separation by electrostatic forces. Through light‐induced photoisomerization, the PC linker was cut off and the NA could release back into the liquid by irradiation with UV light. Thus, functionalized capillaries can be used to separate and concentrate NA. This device allows sample detection by integrating light‐controlled capillaries with a convective system. In addition, an early monitoring and warning platform for infectious diseases was developed based on the IoT for POC testing devices. This system implements many functions, including test result statistics, data visualization, and spread risk warnings. In our future work, this system will incorporate multiple data, such as physical sign detection data, for key populations. Machine learning technology will be used to analyze multi‐source data fusion to provide future forecasts and warnings of pathophoresis. We anticipate that this study will be highly useful for preventing and controlling the propagation of epidemics.

## 4 EXPERIMENTAL METHODS

### 4.1 Materials and chemicals

Streptavidin, 3‐Glycidoxypropyltrimethoxysilane (GPTMS), and bovine serum albumin (BSA) were obtained from Energy Chemical Co., Ltd. The UV‐cleavable oligonucleotide linkers modified with biotin and PCR primer pairs were synthesized by Sangon Biotech (Shanghai) Co., Ltd. The water purification system was bought from ELGA (London, UK). The PCR chemicals, included 10×PCR buffer, dNTPs mixture, and Taq polymerase, were all purchased from TaKaRa Biotechnology (Dalian) Co., Ltd. The fluorescent dye for NA was bought from Invitrogen.

In this work, silica capillaries (1.7 mm i.d. × 3.0 mm o.d.) were purchased from Guanxiu Quartz Co., Ltd. (Lianyungang, China). The semiconductor heater was fabricated by Wenext Co., Ltd. (Shenzhen, China). The temperature controller (REX C100) was obtained from Yixin Sinilink Co., Ltd. (Qingdao, China). The semiconductor heater and temperature sensor were obtained from Haoyi Thermal Electrics Factory (Guangzhou, China). The fluorescence imaging system was bought from Oeabt Optical Technology (China) Co., Ltd.

### 4.2 Bacterial culture

The nutrient broth containing NaCl (0.5 g), beef extract (0.3 g), bacterial peptone (0.5 g) and deionized water (70 mL) was prepared, and the pH value of the broth was adjusted to 7.0. The bacterial strains, including Sta CMCC26003, Lis CMCC54007 and Sal CMCC50040, were obtained from Guangzhou Institute of Microbiology (Guangzhou, China). In orbital shaker, the strains in nutrient broth were cultured at 37°C overnight. We resuspended the bacterium in isotonic saline before experiment.

### 4.3 Acquisition of clinical samples

Throat swab samples from patients infected with COVID‐19 were collected in Shenzhen Third People's Hospital. The studies involving human participants were reviewed and approved by the Shenzhen Third People's Hospital (No. 2020‐0127). All subjects provided written informed consent.

### 4.4 Preparation of light‐controlled capillaries

Before modification, the capillaries were first steeped in piranha solution for 12 h. Next, we used ultrapure water to clean them five times. Then, the cleaned capillaries were dried in pressured gas blowing concentrator at 50°C for 1 h.

The GPTMS solution was first made by dissolving GPTMS (100 μL) in methyl alcohol (9.9 mL). Then, the capillaries were steeped in GPTMS solution for 10 h at room temperature (RT), and the GPTMS coating was formed. The GPTMS‐coated capillary was washed with methylbenzene and ethyl alcohol five times in sequence. Then, the capillaries were dried in the same way as mentioned above.

Subsequently, streptavidin was dissolved in 1× PBS (pH = 7.4), and the final concentration was 1 ng mL^−1^. Then, the GPTMS‐coated capillaries were steeped in the streptavidin solution for 30 min at RT and then stored at 4°C overnight. The 1× PBST solution was used to wash the capillaries five times, and then the capillaries were steeped in BSA (1% in 1×PBS, pH = 7.4) solution overnight at RT to block the redundant sites. The 1×PBST solution was used to clean the uncombined BSA in the capillaries.

The UV‐cleavable oligonucleotide linkers modified with biotin (1 μM) were filled in the capillaries and incubated at RT for 30 min to form the UV‐cleavable layer. The capillaries were cleaned with 1×PBST solution and dried in pressured gas blowing concentrator. Then, the PDDA solution was used to change the potential of the capillaries so that we could use the capillaries to separate NAs by electrostatic forces. The synthesis method was described in our previous work.

### 4.5 Design of the convection PCR device

The complete system included a planiform amplification chamber for convective PCR and a fluorescence signal measuring system. In this work, a planiform thermalization chamber was designed to perform convective PCR. The chamber was made of thermally conductive silicone (34 mm length × 30 mm width × 4 mm depth). The thermal transmissivity of the silicone was 7 W m^−1^ K^−1^. Both ends of the chamber were installed with a heating component (2 cm length × 30 mm width) to keep that side of the chamber at the specified temperature steadily. The chamber was mounted vertically at the bottom of the instrument. When the device was used to perform reverse transcription, both heating components were turned on to keep the reaction area at 50°C. When the device was used to perform convective PCR, the heating component at the bottom end was turned on only, the heat dissipated naturally, and the temperature gradient could be formed in the thermalization chamber. This temperature gradient could be used for simultaneous annealing and extension. Capillaries were installed on the surface of the thermalization chamber. This temperature gradient induced the fluid density deviation between the top and bottom ends, and the reactants could circularly flow between hot and cool zones without active pumping.

The fluorescence signal measuring system is composed of a light source, an optical filter set and a CCD camera. The dominant wavelength of the light source is at 475 nm. The wavelength of the band‐pass excitation filter and emission filter was 455–494 nm and 515–560 nm respectively, and the cutoff wavelength of the dichroic mirror was 501 nm. The CCD camera captured the fluorescence images and the software measured the intensity automatically to produce PCR curves.

### 4.6 Protocols for PCR amplification

The PCR were prepared in a reaction mixture (50 μL) containing PCR buffer (1×), dNTPs (500 μm), PCR primers (4 μm), Taq polymerase (2 unit μL^−1^), 1×SYBR Green (1×) and deionized water. After 50 μL of the mixture was introduced into the capillary, UV light with a wavelength of 365 nm was turned on so that the NA template was released into the liquid. Real‐time PCR was carried out for less than 20 min.

### 4.7 Design of the early monitoring and warning platform

The front‐end and back‐end systems of the early monitoring and warning platform were developed based on the SpringBoot and Vue architecture, respectively. The operation environment is a cloud server with a dual‐core 2 GHz CPU, 16 G memory, 500 G disk and an Ununtn 18.10 and above operation system. JDK 1.8, Mysql 5.7.0, Redis 3.0, Maven 3.6 and other software were preinstalled on the server.

## CONFLICT OF INTEREST STATEMENT

The authors declare no conflicts of interest.

## Supporting information

Supporting Information

## Data Availability

All data related to this study are present in the article. All data associated with this work are available from the corresponding authors upon request.
