# Supplementary material for: Early monitoring‐to‐warning Internet of Things system for emerging infectious diseases via networking of light‐triggered point‐of‐care testing devices
Source: Exploration (Beijing). 2023 Oct 5;3(6):20230028. doi: 10.1002/EXP.20230028 (PMC10742204; doi:10.1002/EXP.20230028)
Supplement: Supplementary file 1 — Supporting Information [file EXP2-3-20230028-s001.docx]

Supporting Information

**Early Monitoring-to-Warning Internet-of-Things System for Emerging Infectious Diseases via Networking of Light-Triggered Point-of-Care Testing Devices**

*Yu Fu^2,4†^, Yan Liu^6†^, Wenlu Song^1†^, Delong Yang^5†^, Wenjie Wu^4†^, Jingyan Lin^4^, Xiongtiao Yang^2^, Jian Zeng^2^, Lingzhi Rong^2^, Jiaojiao Xia^2^, Hongyi Lei^2*^, Ronghua Yang^3*^, Mingxia Zhang^4*^, Yuhui Liao^1*^*

**1. Sequence of oligonucleotide primer pairs**

The sequences of oligonucleotide primer pairs used in the experiments are list in **Table S1** which were synthesized by Sangon Biotech (Shanghai) Co., Ltd.

**Table S1.** Oligonucleotide Sequences

| **oligonucleotide** | **sequence (5’−3’)** |
| --- | --- |
| primer-F for *Sta* | GGTCCTGAAGCAAGTGCATT |
| primer-R for *Sta* | ATACGCTAAGCCACGTCCAT |
| primer-F for *Sal* | TGCTCAGACATGCCACAGT |
| primer-R for *Sal* | TGCTCGTAATTCACCACCATTG |
| primer-F for *Lis* | CAAGTCCTAAGACGCCAATC |
| primer-R for *Lis* | CAAGTCCTAAGACGCCAATC |
| primer-F for COVID-19 | GACCCCAAAATCAGCGAAAT |
| primer-R for COVID-19 | TCTGGTTACTGCCAGTTGAATCTG |
| Target of COVID-19 | GACCCCAAAAUCAGCGAAAUGCACCCCGCAUUACGUUUGGUGGACCCUCAGCUUCAACUGGCAGUAACCAGA |

**2. Function Introduction of the Early Monitoring and Warning Platform**

**2.1 System management**

*Subscriber management*


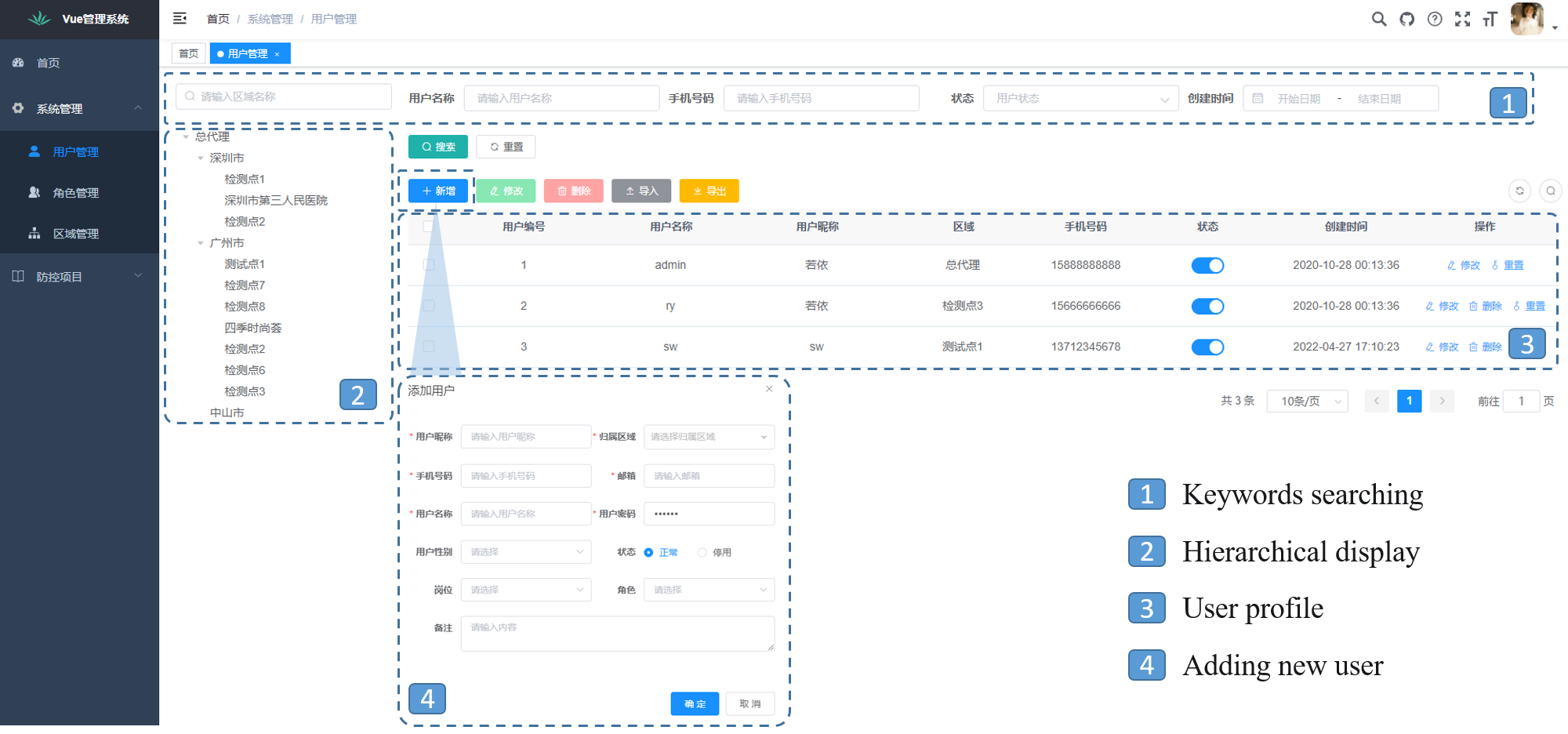


**Figure S1.** Subscriber management interface of early monitoring and warning platform

The subscriber management interface is shown in Figure S1, through this board, the platform can add users, including platform users, detection personnel, management personnel and equipment maintenance personnel at detection points, and their information about name, area, phone number, mailbox, password, etc.

*Role Management*

As shown in Figure S2, the role management module is used to manage the permissions of registered personnel, including the internal sub permissions of the management system, such as adding and deleting personnel information, changing management permissions, etc.; and control system authority, such as equipment using authority, equipment maintenance authority, consumables application authority, etc. The specific adding method is shown in Figure S2 B, we can enter the role name, permission character and role order, confirm status (normal or disabled) and menu permissions (system management or prevention and control project) to complete this step.

**
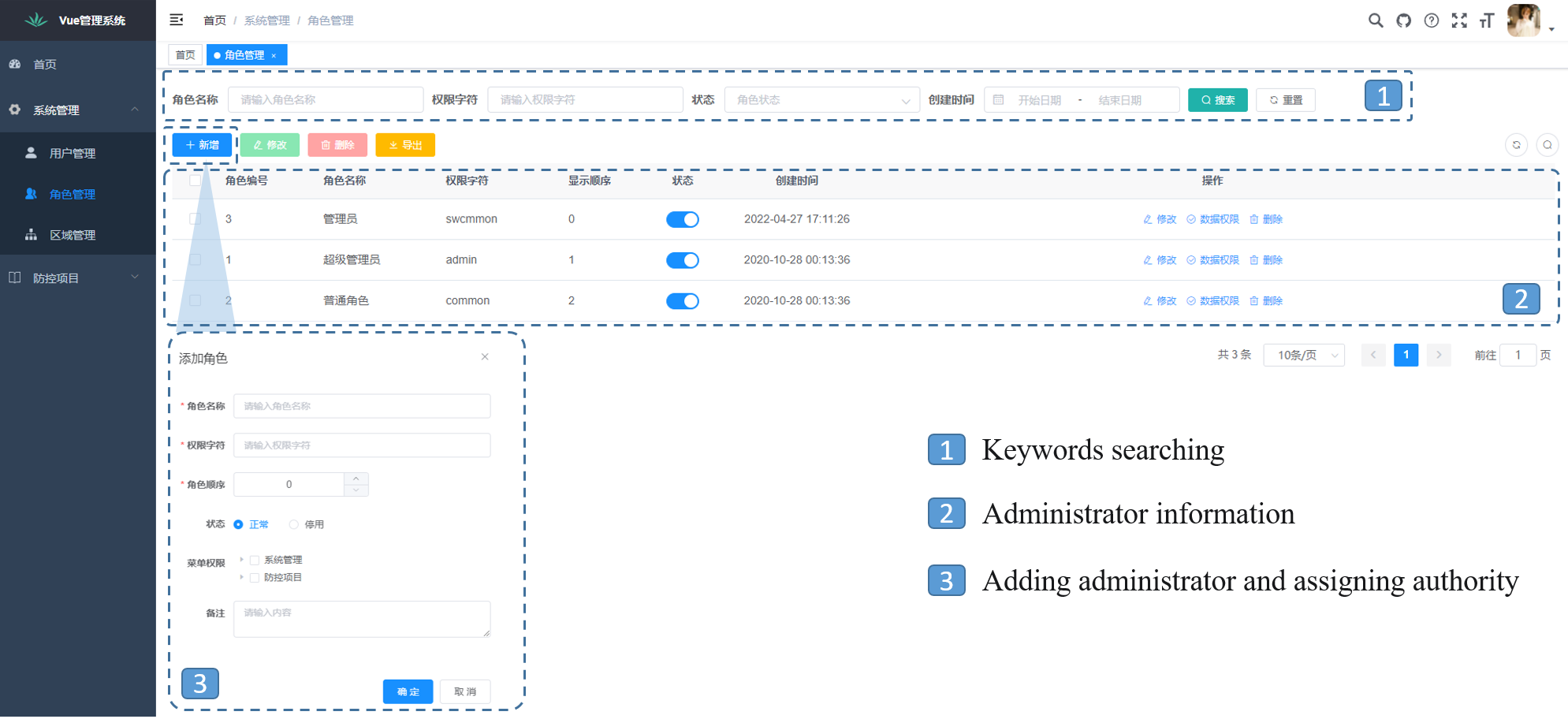
**

**Figure S2.** Role management interface of early monitoring and warning platform

*Regional Management*

As shown in Figure S3, the regional management module is mainly used to add, to delete and display the established detection point information, including longitude and latitude, address, working status, creation time, etc.; the specific adding method is shown in Figure S3 B, we can confirm the superior area, and enter area name, sort, person in charge, contact number, email, etc. of the selected area to complete this step.

**
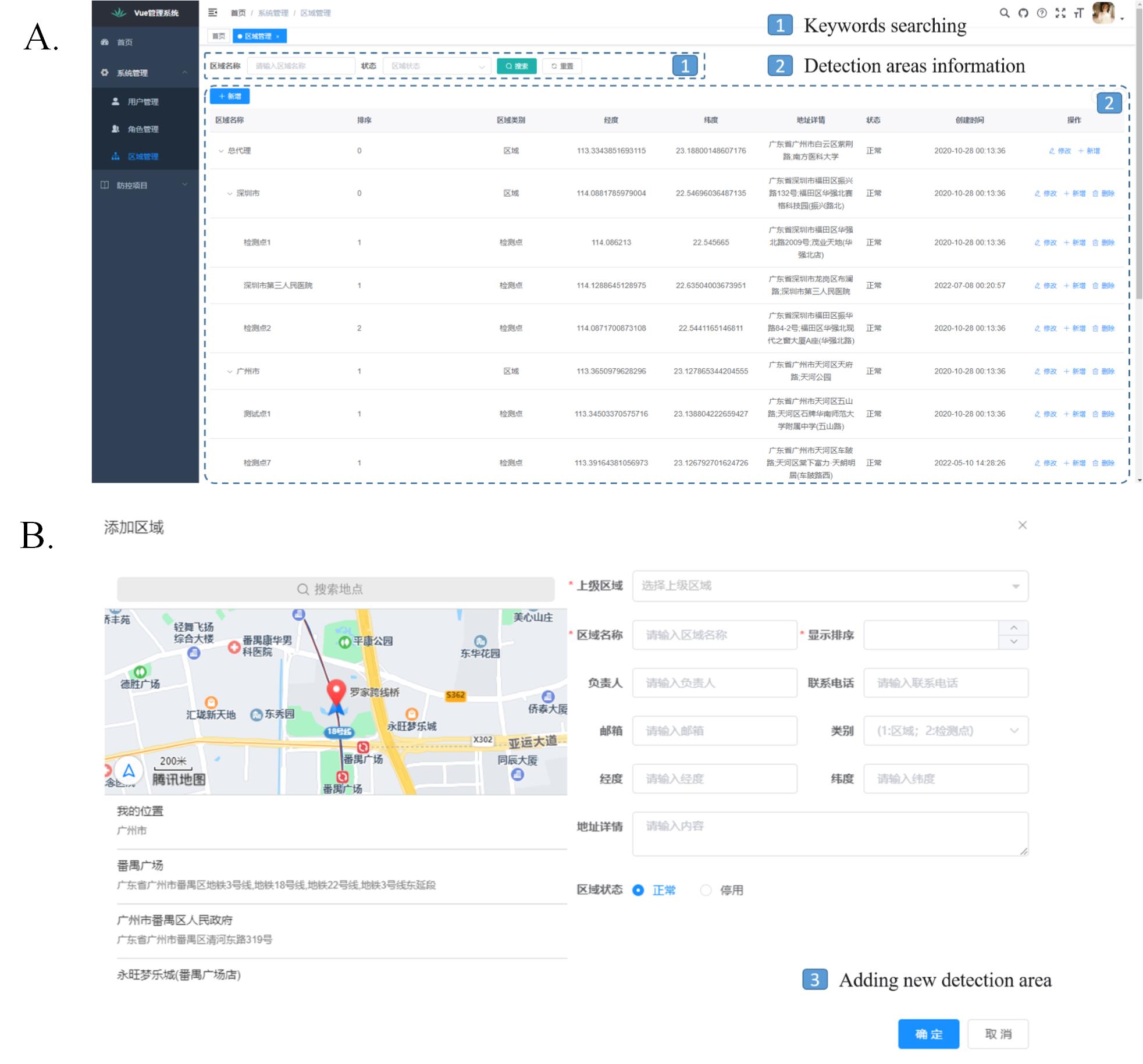
**

**Figure S3.** Regional management interface of early monitoring and warning platform

**2.2 Information management**

*Risk regions*

Risk regions management is shown in Figure S4, through this board, the platform can add, delete, display risk areas at different levels. The specific adding method is shown in Figure S4 B, we can confirm the name, longitude, latitude, level, status (normal or disabled) of the risk region to complete this step.

**
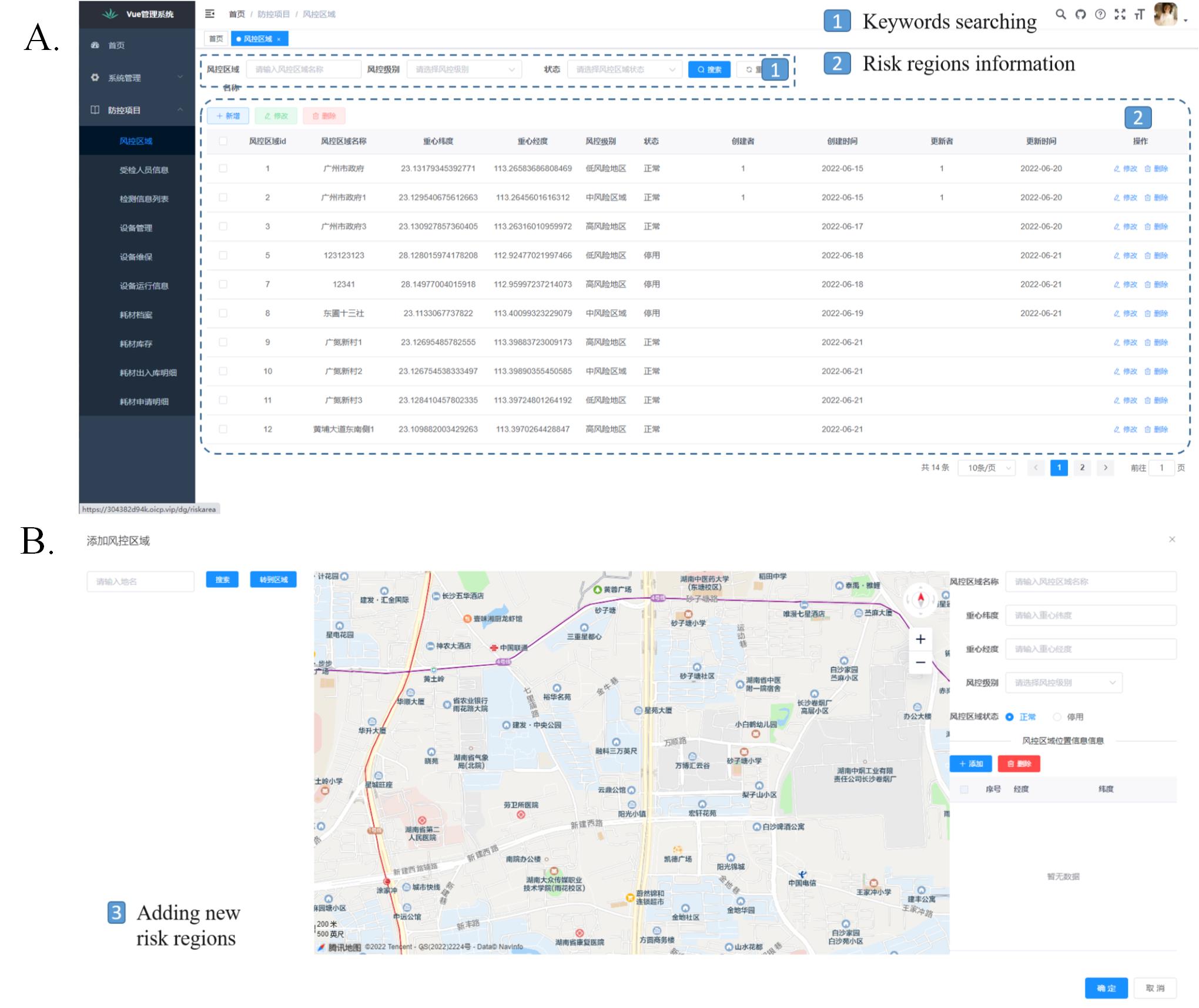
**

**Figure S4.** Risk regions management interface of early monitoring and warning platform

*Information of the persons to be tested*


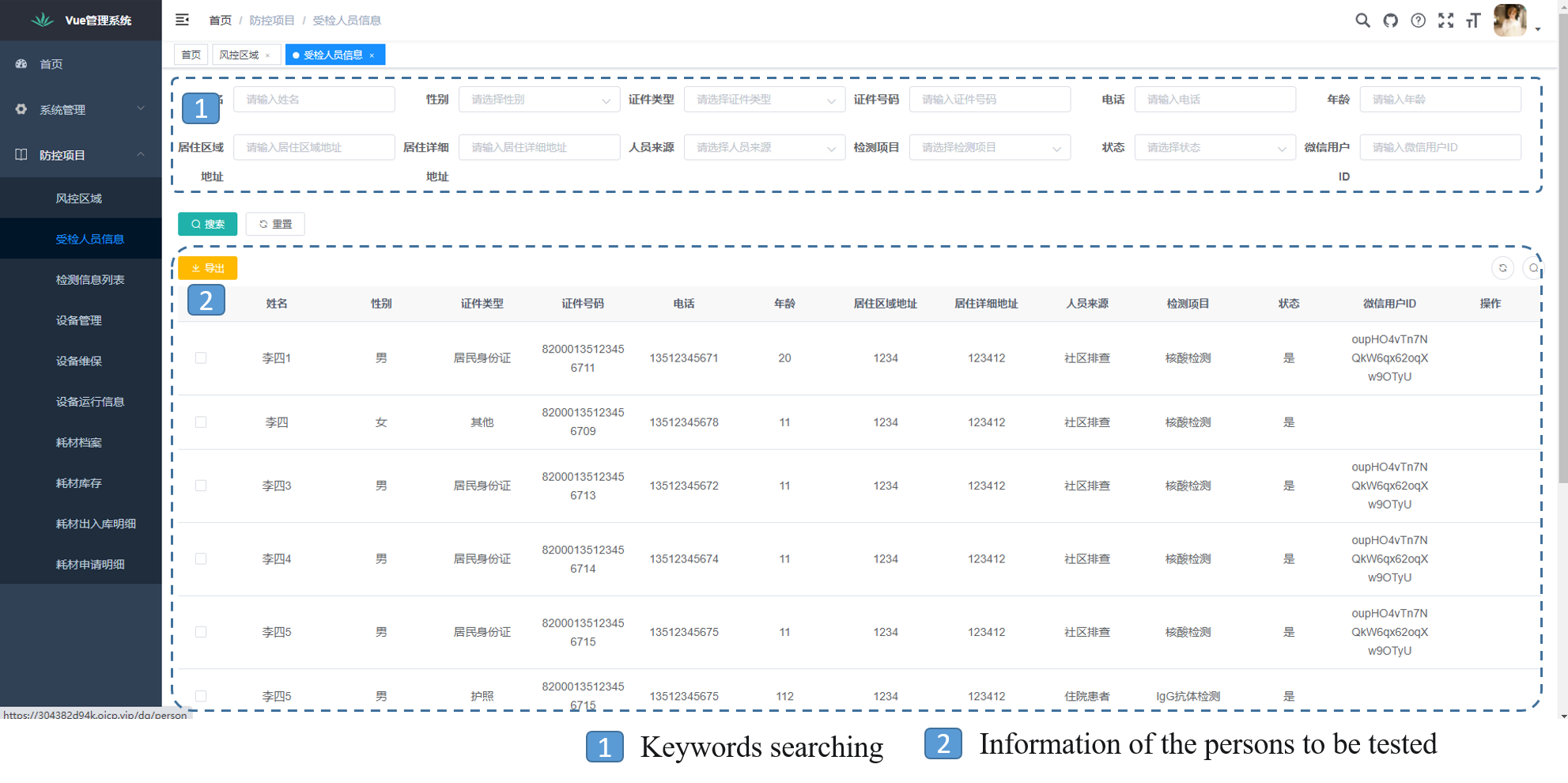


**Figure S5.** The interface of information of the persons to be tested

As shown in Figure S5, the information of the persons to be tested can be queried in this interface, including name, gender, certificate type, certificate number, telephone number, age, residential address, personnel type, test items, etc.

*Detection results*


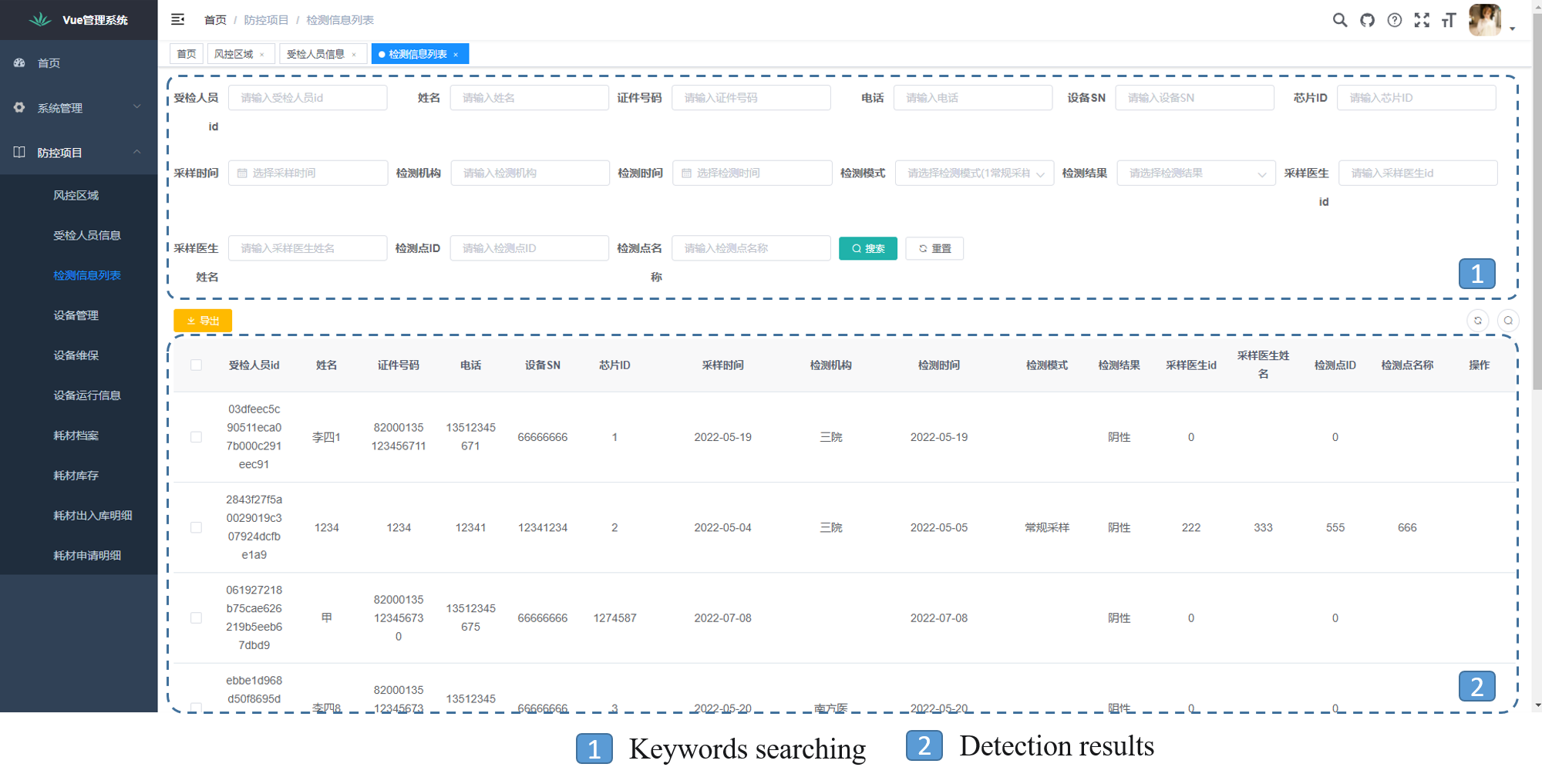


**Figure S6.** The interface of detection results

As shown in Figure S6, by entering different key words, such as name, certificate number or telephone number, etc., the detection results can be queried in this interface, including name, certificate number or telephone number, sampling time, testing organization, testing time, testing results, etc.

*Equipment management*

As shown in Figure S7, the equipment information of each detection area can be queried and managed in this interface, the specific adding method is shown in Figure S7 B, we can enter the SN of equipment, ID, name, status of detection area to complete this step.

**
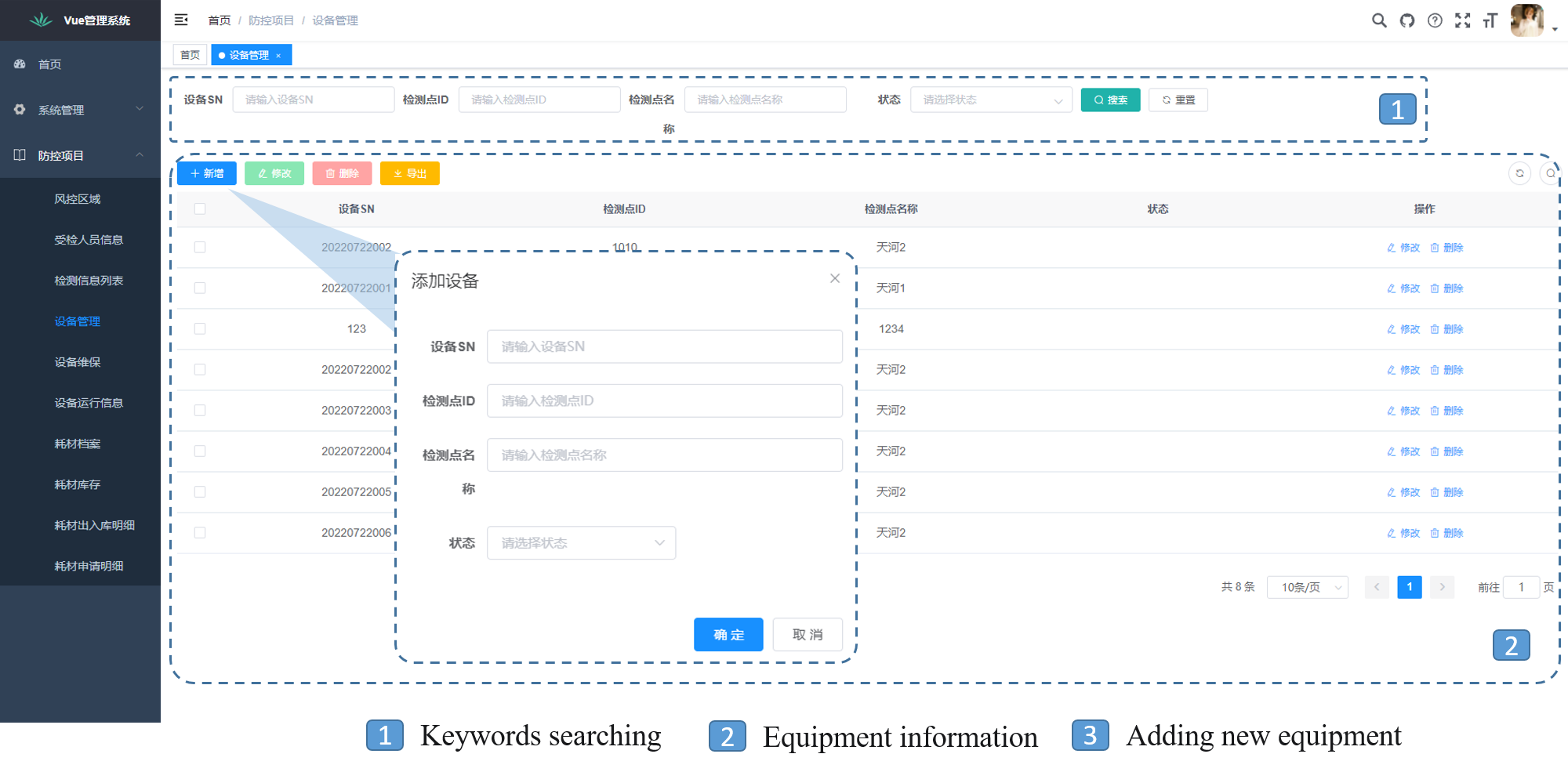
**

**Figure S7.** The interface of equipment management

*Facilities maintenance*

As shown in Figure S8, facilities maintenance information can be queried in this interface, including the SN of equipment, submission time, the ID and name of detection area, and type, time, content, result of the facilities maintenance, etc.


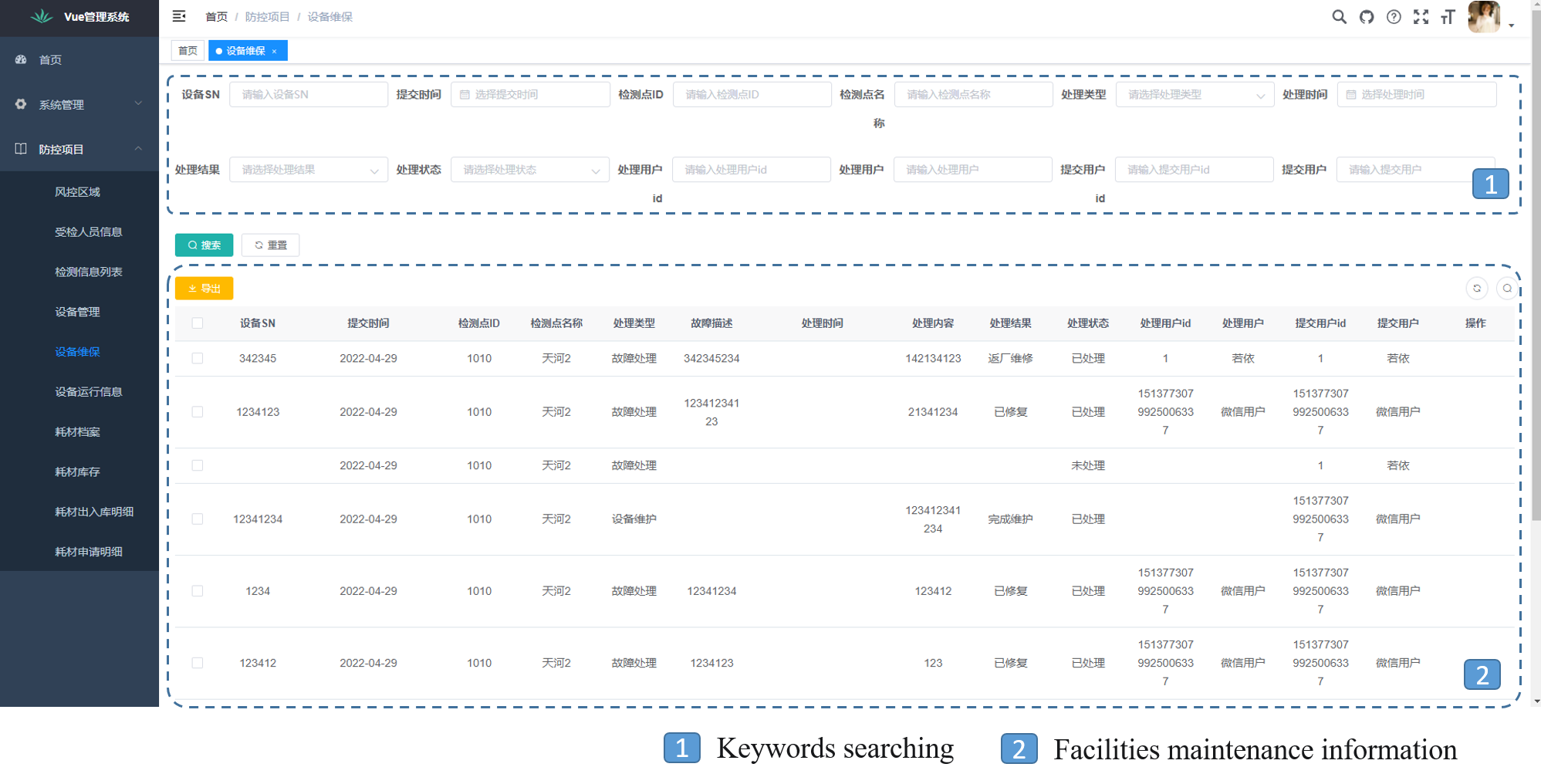


**Figure S8.** The interface of facilities maintenance management

*Facilities operation*


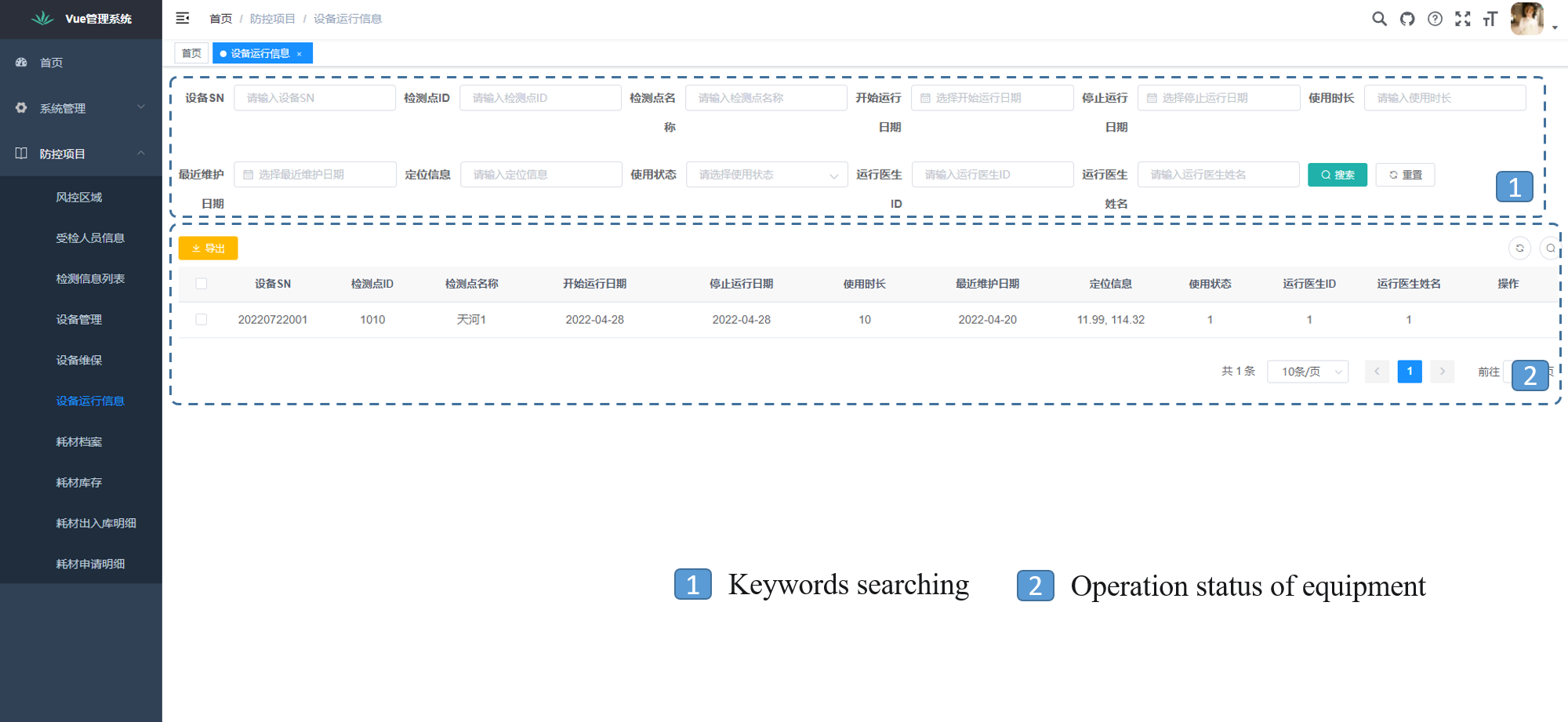


**Figure S9. T**he interface of facilities operation

As shown in Figure S9, the operation status of detection equipment can be queried in this interface, including the SN of equipment, start operation time, end operation time, operation duration, latest maintenance date, positioning information and the ID and name of the operator, etc.

*Consumable information*


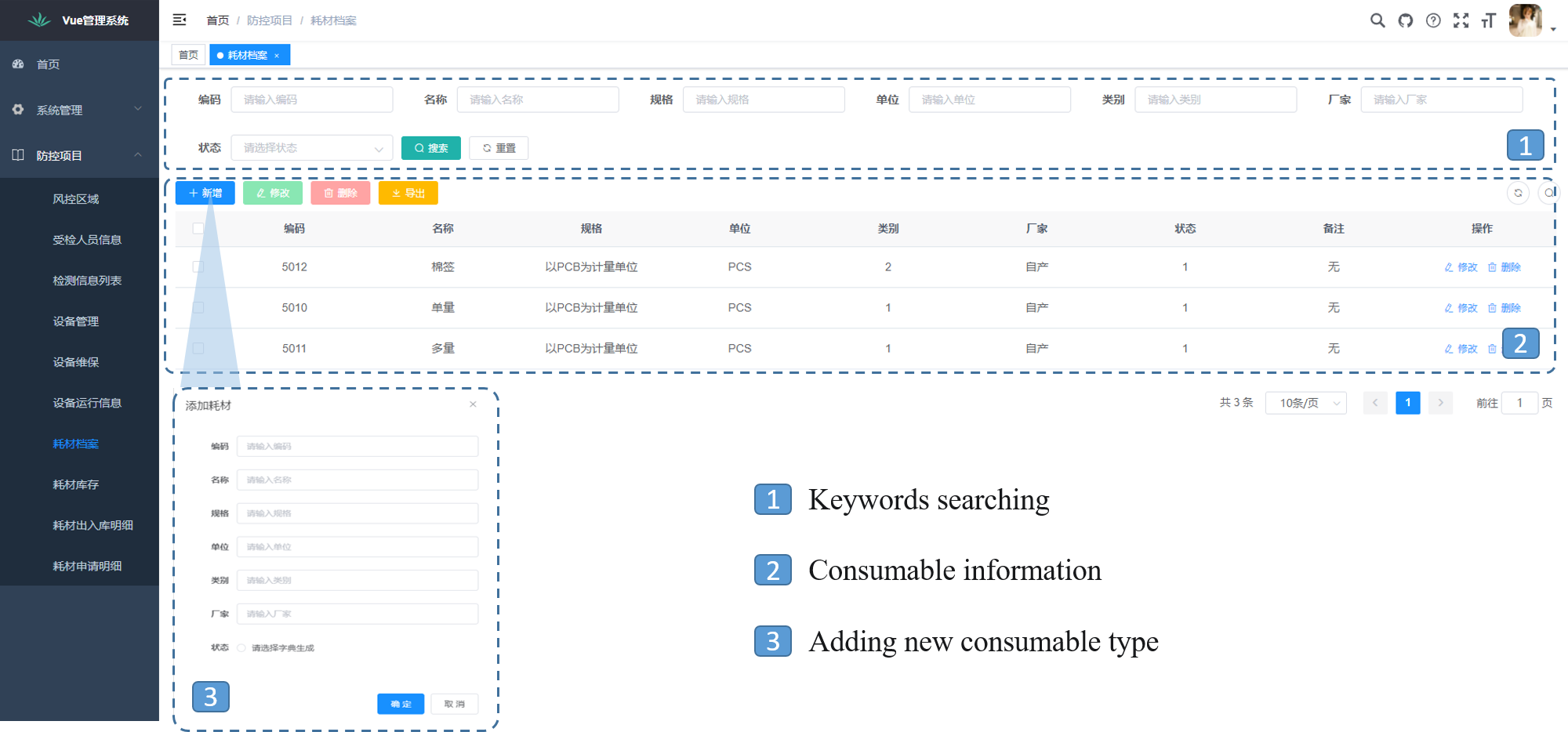


**Figure S10.** The interface of consumable information

As shown in Figure S10, the consumable information can be queried in this interface, including code, name, specification, unit, type (swabs, capillary, single channel detection chip, multi-channel detection chip), manufacturer, etc. The specific adding method is shown in Figure S10 B, we can enter the code, name, specification, unit, type, manufacturer of consumable materials to complete this step.

*Consumable inventory*

**
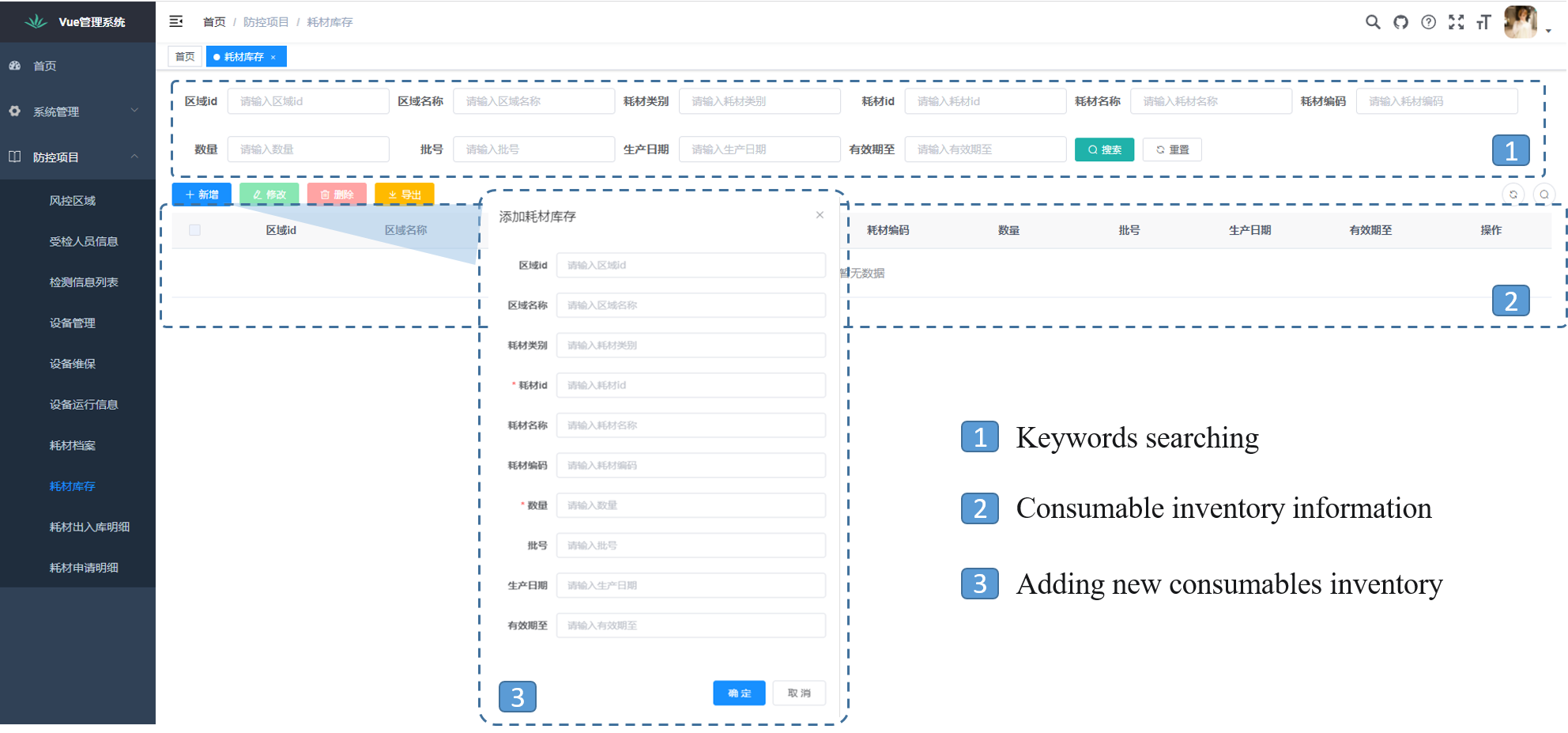
**

**Figure S11.** The interface of consumable inventory

As shown in Figure S11, the consumable inventory can be queried in this interface, including the ID and name of area, the type, ID, name, code, quantity, batch number, production date and expiration date of consumable materials. The specific adding method is shown in Figure S11 B, we can enter the above information to complete this step.

*Material storage details*

**
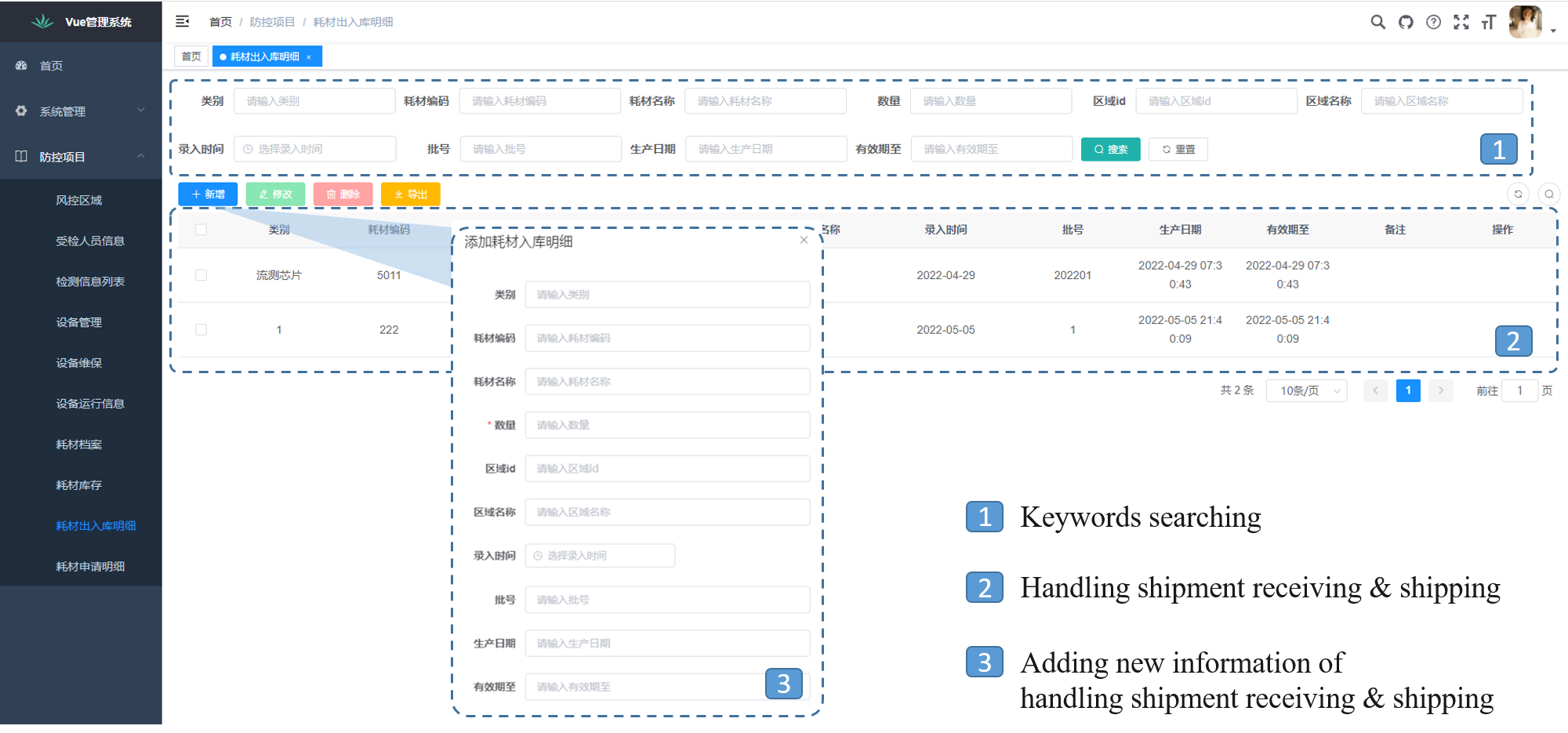
**

**Figure S12.** The interface of material storage details

As shown in Figure S12, the material storage details can be queried in this interface, including type, code, name, quantity, warehousing time, batch number, production date and expiration date of material, the ID name of the area, as shown in Figure S12 B, we can enter above information to complete warehousing registration.
